# Genetic regulation of the *ramA* locus and its expression in clinical isolates of *Klebsiella pneumoniae*

**DOI:** 10.1016/j.ijantimicag.2011.02.012

**Published:** 2011-07

**Authors:** R. Rosenblum, E. Khan, G. Gonzalez, R. Hasan, T. Schneiders

**Affiliations:** a Centre for Infection and Immunity, 4th Floor, Medical Biology Centre, 97 Lisburn Road, Belfast BT9 7BL, UK; b Department of Pathology and Microbiology, Aga Khan University, Stadium Road, P.O. Box 3500, Karachi 74800, Pakistan; c Laboratorio de Investigación en Antibióticos, Departamento de Microbiología, Facultad de Ciencias Biológicas, Universidad de Concepción, Barrio Universitario s/n. Concepción, Chile

**Keywords:** *Klebsiella pneumoniae*, *romA*, *ramA*, *ramR*, *acrA*, Tigecycline

## Abstract

Tigecycline resistance has been attributed to *ramA* overexpression and subsequent *acrA* upregulation. The *ramA* locus, originally identified in *Klebsiella pneumoniae*, has homologues in *Enterobacter* and *Salmonella* spp. In this study, we identify in silico that the *ramR* binding site is also present in *Citrobacter* spp. and that *Enterobacter*, *Citrobacter* and *Klebsiella* spp. share key regulatory elements in the control of the *romA*–*ramA* locus. RACE (rapid amplification of cDNA ends) mapping indicated that there are two promoters from which *romA*–*ramA* expression can be regulated in *K. pneumoniae*. Correspondingly, electrophoretic binding studies clearly showed that purified RamA and RamR proteins bind to both of these promoters. Hence, there appear to be two RamR binding sites within the *Klebsiella romA*–*ramA* locus. Like MarA, RamA binds the promoter region, implying that it might be subject to autoregulation. We have identified changes within *ramR* in geographically distinct clinical isolates of *K. pneumoniae.* Intriguingly, levels of *romA* and *ramA* expression were not uniformly affected by changes within the *ramR* gene, thereby supporting the dual promoter finding. Furthermore, a subset of strains sustained no changes within the *ramR* gene but which still overexpressed the *romA*–*ramA* genes, strongly suggesting that a secondary regulator may control *ramA* expression.

## 1 Introduction

*Klebsiella pneumoniae* is a major nosocomial pathogen that causes both community- and hospital-acquired infections. Strains with chromosomal and/or plasmid-mediated resistance mechanisms coupled with efflux/influx-related mutations are being increasingly identified [1–3]. The continued antimicrobial challenge of *K. pneumoniae* has precipitated the emergence of clones harbouring a plethora of resistance mechanisms to clinically relevant antibiotics (e.g. fluoroquinolones, third-generation cephalosporins and carbapenems) and emerging pandrug-resistant clones have left few therapeutic strategies available to combat this pathogen [4].

Tigecycline is a new glycylcycline with substantial anti-Gram-negative activity that has been introduced for the treatment of community-acquired Gram-negative infections caused by extended-spectrum β-lactamase-producing *K. pneumoniae* and *Escherichia coli* [5]. A recent study has shown that tigecycline is effective against pandrug-resistant *K. pneumoniae* and *Enterobacter* spp. Tigecycline is able to evade the classical mechanisms that confer resistance to tetracycline and minocycline, such as Tet(A–E)-mediated efflux and ribosomal protection conferred by Tet(M) [5]. However, tigecycline appears to be vulnerable to efflux by chromosomally encoded efflux pumps [6–10].

Studies into tigecycline resistance in members of the Enterobacteriaceae have shown that it is mediated via upregulation of efflux pumps that are controlled by certain regulatory loci [6–8,10]. For instance, several studies have demonstrated that tigecycline resistance results from upregulation of AraC family transcriptional regulators such as MarA or RamA, which in turn are linked to increased expression of the AcrAB efflux pump [6,7,10,11]. This pump not only functions as a clinically relevant drug exporter but has also been demonstrated to affect significantly the virulence potential of the bacterium [12,13]. The AcrAB efflux pump is locally controlled by the transcriptional repressor AcrR [14]. However, transcription factors such as RamA and MarA are able to override AcrR-mediated repression and upregulate expression of the AcrAB efflux pump [6,7,10,11]. Accordingly, strains with wild-type AcrR and increased RamA or MarA expression are linked to increased AcrAB levels [11]. Whether levels of AcrA upregulation in the absence of *acrR* mutations are as significant as those seen when *acrR* is mutated is not clear.

RamA is a member of the AraC/XylS family and is closely related to the MarA and SoxS proteins [15]. The *ramA* locus is only found in *Klebsiella*, *Salmonella*, *Enterobacter* and *Citrobacter* spp.; the genetic organisation in *Salmonella* differs from the others owing to lack of the *romA* gene. Like MarA, increased *ramA* expression is linked to upregulation of the AcrAB efflux pump, which confers a multidrug-resistant phenotype to a variety of different antibiotic classes [7,10,11,16]. This phenotype is mediated exclusively through the *acrAB* gene, as *ramA* overexpression in an *acrAB*-deleted strain does not produce a similar phenotype [11].

**Fig. 1:**
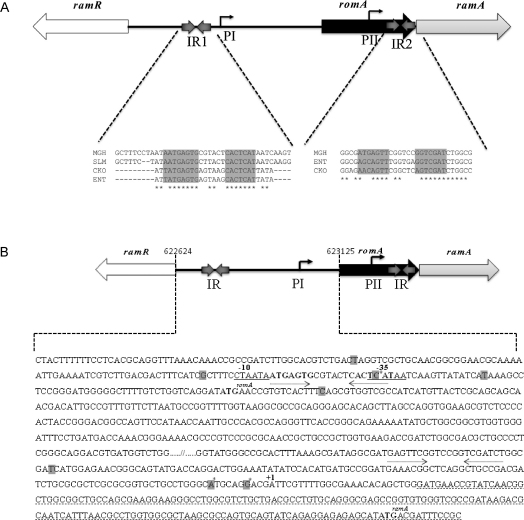
(A) Alignment of the RamR palindromic binding sequence at the pI and pII promoter in *Salmonella*, *Klebsiella*, *Enterobacter* and *Citrobacter* spp. (B) Regulatory elements within the *romA*–*ramA* locus. The inverted palindromic sequences are in bold and indicated by arrows. The two putative promoters are depicted as pI and pII. The transcription start of *ramA* as mapped by 5′ RACE (rapid amplification of cDNA ends) is indicated by +1. The translation start site of both *romA* and *ramA* is indicated in bold and superscript. All promoter changes are in grey, with those hypothesised to be associated with *ramA* overexpression in grey and with an asterisk. IR, intergenic region.

Expression of the *ramA* gene is controlled at a transcriptional level. Studies both in *Salmonella enterica* serovar Typhimurium [17] and *K. pneumoniae* [16] have identified a *tetR*-like gene, called *ramR*, that lies upstream of the *ramA* gene and acts as its repressor (Fig. 1). Of note, the stop codon of the *ramR* gene overlaps the start codon of the *ybdF* gene, implying that regulation of these genes is very likely linked. Studies both into *S.* Typhimurium [17] and *K. pneumoniae* [16] have reported that *ramR* mutations are directly linked to *ramA* overexpression. Both in *Salmonella* and *Klebsiella*, bioinformatic analyses suggest that the RamR protein binds a palindromic sequence that is either overlapping or downstream of the –10 sequence of the *ramR* gene [16,17]. In *K. pneumoniae* and *Enterobacter* spp., the genomic organisation of *ramR*, its corresponding palindromic binding sites and *ramA* is conserved compared with *Salmonella*, although these bacteria also harbour the *romA* gene (Fig. 1). Mutations within the *ramR* gene have been shown to result in *ramA* upregulation [16,17], however the effect of this derepression on the surrounding genes within the locus, i.e. *romA*, is not known.

In studies involving clinical strains, *ramA* upregulation is not normally the sole mechanism of resistance but appears to act in combination with other mutations, e.g. cefuroxime-resistant isolates also showed a decrease in levels of the outer membrane protein OmpK35 [18], and in fluoroquinolone-resistant isolates upregulation of the *ramA* gene was in association with target-specific mutations in the topoisomerase genes (e.g. *gyrA* and *parC*) and mutations within *acrR,* the repressor of the *acrAB* efflux pump [11]. Hence, the aims of this study were to elucidate the genetic regulation of the *ramA* locus and whether its overexpression is always linked to changes within the *ramR* gene.

## 2 Materials and methods

### 2.1 Clinical isolates

All isolates were identified as *K. pneumoniae* and were isolated from intensive care wards in hospitals within Chile, Turkey and Pakistan from 2006–2008. Three strains from Singapore characterised in a previous study [11] as well as the original strains (Ecl8 and Ecl8Mdr1) described by George et al. [15] were also included. Other strains used as controls in this study were: for validation of the AcrA Western blot analyses, AG100A (deleted for *acrA*) and AG100B (deleted for *acrR*, AcrA-overexpresser); and for validation of the *ramA* reverse transcription polymerase chain reaction (RT-PCR), TS67 (deleted for *ramA*) and TS68 (deleted for *ramR*) [19]. The mutants TS67 and TS68 were generated from *K. pneumoniae* Ecl8 using a modified protocol as described by Merlin et al. [20].

### 2.2 Minimum inhibitory concentration (MIC) testing

MICs of tigecycline (gift from Wyeth Pharmaceuticals) for the clinical isolates were determined and interpreted according to British Society for Antimicrobial Chemotherapy (BSAC) protocols [21]. Strains exhibiting tigecycline MICs ≤1 μg/mL and >2 μg/mL were classed as sensitive and resistant, respectively.

### 2.3 Mapping the transcriptional start site of the ramA locus

The transcriptional start site of the *ramA* locus was mapped according to the manufacturer's instructions using the 5′ RACE (rapid amplification of cDNA ends) system (Invitrogen, Carlsbad, CA). Briefly, 0.5 μg of RNA extracted at mid log phase was reverse transcribed into first-strand cDNA, which was then terminal deoxynucleotidyl transferase (TdT)-tailed. Subsequently, the TdT-tailed cDNA was amplified by PCR and was assessed for positive PCR products. A second round of amplification using nested gene-specific primers (Table 1) and Abridged Universal Amplification Primer (AUAP) was performed prior to the resulting products being subcloned into pGEM^®^-T Easy vector (Promega, Southampton, UK) for sequencing.

### 2.4 Gene expression analyses using RT-PCR

**Fig. 2:**
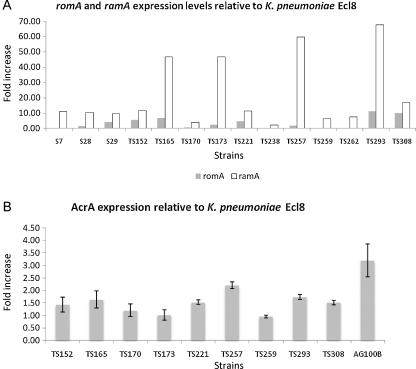
(A) Bar chart showing levels of *romA* and *ramA* expression in *Klebsiella pneumoniae* clinical strains. Levels of *romA* and *ramA* were normalised to *K. pneumoniae* Ecl8 (*ramA* non-expresser). (B) Bar chart showing levels of AcrA expression. Relative fold increases in the AcrA levels were quantified after comparisons with a wild-type sensitive *K. pneumoniae* isolate Ecl8.

RT-PCR was performed to assess the expression levels of the *ramA* and *romA* genes. RNA samples were extracted from the different clinical isolates at mid log phase [optical density at 600 nm (OD_600_) 0.5–0.8] using TRIzol^®^. Total RNA was digested with DNase I to ensure the removal of contaminating genomic DNA prior to cDNA synthesis. For cDNA synthesis, a SuperScript^®^ VILO™ cDNA Synthesis Kit (Invitrogen) was used. Briefly, 300 ng of DNase I-treated total RNA was converted to cDNA and was used in PCRs with gene-specific primers shown in Table 1. A 1 in 10 cDNA dilution was used for amplification of the 16S gene. The resulting PCR products were subjected to electrophoresis on a 1.5% agarose gel. Densitometric analyses using Multi Gauge FujiFilm software of the gel bands were performed and normalised to 16S expression. The fold increase in *ramA* expression relative to the sensitive strain Ecl8 (*ramA* non-expresser) is shown in Fig. 2A.

### 2.5 Electrophoretic mobility shift assay (EMSA)

The *romA*–*ramA* promoter regions were amplified and subjected to EMSA with both purified RamA and RamR proteins. Purified RamA and RamR proteins were extracted from recombinant pET constructs containing the *ramA* and *ramR* genes using metal chelation chromatography on nickel/nitrilotriacetate superflow agarose (QIAGEN, Crawley, UK). Briefly, end-labelled (using [γ-^32^P] ATP; Perkin Elmer, Boston, MA) PCR products were incubated with increasing concentrations (200 nM and 400 nM) of RamA or RamR in binding buffer (125 mM Tris–Cl, 250 mM KCl, 5 mM dithiothreitol [DTT], 160 ng of salmon sperm DNA and 25% glycerol). The complexes were run on 5% native polyacrylamide gel electrophoresis (PAGE) gels for 2.5 h. The gel was then dried and exposed to the phosphor screen for image analysis. To confirm that the interactions between RamA or RamR and the promoter regions were specific, competition experiments with bovine serum albumin (BSA) as a negative control and with cold promoter were also performed.

### 2.6 Mutations within the promoter regions and the ramR and acrR genes

For those strains where *ramA* expression was elevated, the *ramR* and *acrR* genes and the pI/pII promoter regions were amplified using the primers shown in Table 1. The BigDye™ reaction (Applied Biosystems, Warrington, UK) was set up prior to the products being sequenced at the Genomics Core Facility in Belfast City Hospital (Belfast, UK).

### 2.7 Western blot analyses

Levels of AcrA were determined by Western blot analyses as described previously by Schneiders et al. [11]. Briefly, cultures were grown to mid log phase (OD_600_ ca. 0.6–0.7) prior to protein extraction using sonication. Then, 15 μg of total protein was loaded onto a 10% NuPAGE^®^ gel (Invitrogen) prior to transfer to a nitrocellulose membrane as per the manufacturer's instructions. The blots were hybridised with the primary antibody anti-AcrA (kind gift from H. Zgurskaya, University of Oklahoma, Norman, OK) (1:40 000) overnight at 4 °C. The secondary antibody IRDye-800 goat anti-rabbit IgG (Li-Cor Biosciences, Lincoln, NE) was incubated with the blots for 1 h at room temperature and the membranes were scanned using the Odyssey Infrared Imaging system, which is based on near infrared fluorescence detection. The membrane was scanned in two channels at 700 nM and 800 nM, which detects the green and red channels, respectively. All images were quantified using the reading from the 800 nM channel. Analysis of band intensities to assess AcrA levels was performed using the Odyssey software v3 (Li-Cor Biosciences). Levels of AcrA protein of all test strains were compared against the sensitive *K. pneumoniae* strain Ecl8 to report a relative fold increase in AcrA levels. Both AG100A and AG100B were also used as controls in the AcrA Western blots.

## 3 Results

### 3.1 Minimum inhibitory concentrations

The MIC_50_ and MIC_90_ values (MICs for 50% and 90% of the organisms, respectively) of tigecycline, minocycline, tetracycline, ciprofloxacin and ceftazidime against the strains are shown in Table 2. Of the 157 strains tested, 24 were resistant to tigecycline based on the BSAC breakpoint criteria [21]. MICs to tigecycline generally ranged from 0.25 μg/mL to 4 μg/mL. Minocycline and tetracycline MIC_90_ values were 128 μg/mL and >128 μg/mL, respectively; however, cross-resistance to tigecycline was not observed, consistent with previous survey data [22]. Tigecycline-resistant isolates exhibited cross-resistance to ceftazidime and ciprofloxacin.

### 3.2 Bioinformatic analyses for RamR binding sites in *Klebsiella pneumoniae* (accession no. NC_009648), Citrobacter koseri (accession no. NC_009648) and Enterobacter sp. 638 (accession no. NC_009648)

In S. Typhimurium, *ramR* has been shown to function as its cognate negative regulator [17]. Given the similarities in the genomic organisation of the *ramR* and *romA*–*ramA* loci between *Klebsiella*, *Enterobacter*, *Citrobacter* and *Salmonella*, we sought to confirm whether *ramR* controlled the expression of the *romA*–*ramA* locus directly in *Klebsiella* and whether other accessory binding sites existed for RamR within the *romA–ramA* locus. Bioinformatic analyses using ClustalW (http://www.ebi.ac.uk/Tools/clustalw2/index.html) showed that the palindromic binding sites identified for RamR in *Salmonella* spp. are conserved within the intergenic region between the *ramR* and *romA* coding sequences of *K. pneumoniae*, *Enterobacter* and *Citrobacter* spp. (Fig. 1A). There appeared to be no perfect palindromic site for RamR upstream of the *ramA* gene. Thus, we surmised that regulation of the *romA*–*ramA* locus mediated by RamR must occur via the palindromic sequence that lies upstream of the *romA* gene.

### 3.3 RACE mapping

The transcriptional start site of *ramA* as determined by RACE mapping is shown in Fig. 1B. The transcriptional start site (hereby called pII) (Fig. 1B) for *ramA* was found upstream of the *romA* stop codon, which implies that *romA* and *ramA* are part of an operon and are likely co-transcribed in *K. pneumoniae*. The transcriptional start site mapped corresponds with our previous observation that two transcripts sized ca. 0.6 kb and ca. 0.9 kb were obtained with Northern blotting. The larger transcript must correspond to the promoter that lies upstream of the *romA* gene. The presence of the pII promoter also suggests that *ramA* expression can be modulated independently of *romA*. Correspondingly, this differential regulation is evident in the differing levels of transcription of the *romA* and *ramA* genes as shown in Fig. 2A.

### 3.4 Electrophoretic mobility shift assay

**Fig. 3:**
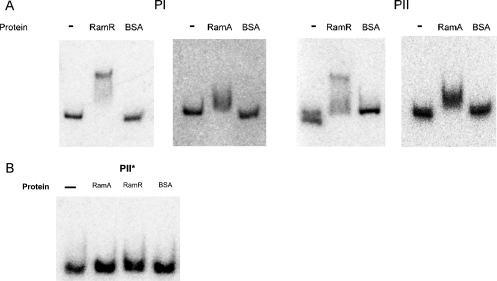
Electrophoretic mobility shift assay (EMSA) of the promoter regions with purified RamA or RamR proteins. (A) EMSA showing the binding of purified RamR and RamA to the pI and pII promoter regions. (B) pII* represents the region underlined (dashed lines) in Fig. 1B.

Bioinformatic analyses indicate the presence of a putative but conserved palindromic binding site for RamR within the pI region. However, no such site could be observed for the pII promoter. We also wondered whether RamA, like other similar regulators (i.e. MarA), autoregulates itself by binding to its own promoter region. The intergenic regions, designated pI and pII, bound both purified RamR and RamA proteins (Fig. 3A). The interactions of the pI and pII promoter regions with the RamA and RamR proteins were found to be specific, as no shifts were observed with BSA alone (Fig. 3A) and addition of cold promoter was able to reduce binding of the proteins to the labelled probe (data not shown). Of note, the fragment of DNA (pII*) that contained no transcriptional signals was found not to bind either RamA or RamR (Fig. 3B). As indicated previously, the pI promoter has a conserved RamR binding site that is required for RamR-mediated control. However, the lack of a similar or identical RamR binding site (by bioinformatic analyses) indicates that RamR-mediated regulation of the pII promoter may be mediated via a more degenerate palindromic binding site. Accordingly, bioinformatic analyses using the EMBOSS Pairwise Alignment Tool (http://www.ebi.ac.uk/Tools/emboss/align/) of the pII promoter region implies that there is a putative but degenerate palindromic site upstream of the *ramA* gene (Fig. 1A).

### 3.5 DNA mutations in the promoter regions pI and pII and the ramR and acrR genes

#### 3.5.1 Mutations within the ramR gene and levels of romA and ramA expression

Of the 24 strains that were resistant or intermediately susceptible to tigecycline, only 10 harboured mutations within the *ramR* gene. Changes within the *ramR* gene were found in both the DNA- and ligand-binding domains of the protein (prediction based on PSIPREDv3 [23]), suggesting that there are no mutational hotspots within *ramR*, although the majority of changes were clustered within the ligand-binding domain. As previously suspected, not all of the changes observed within RamR resulted in *ramA* expression, e.g. changes H186N and A187E observed in TS202 and change E41K in TS215 did not result in *ramA* overexpression. The change I141T was observed in four of the isolates tested (Ecl8, Ecl8Mdr1, TS262 and TS293) (Table 3A). Since this change is also found in the sensitive *Klebsiella* strain Ecl8 it is likely that it is not associated with *ramA* overexpression. In addition, strains TS173, TS221, TS257 and TS259 did not harbour changes within the *ramR* gene (Table 3A) and/or the intergenic regions but still overexpressed *ramA* (Fig. 2A). Several strains exhibiting MICs ≥ 4 μg/mL were found to overexpress *ramA* but not to harbour any changes within the *ramR* gene. Interestingly, levels of *ramA* expression between those strains that harboured changes within *ramR* were not higher than those strains that sustained no changes within the gene. In addition, transcriptional levels of the *romA* gene did not appear to be linked to *ramA* expression (Fig. 2A), although increased expression of *romA* was generally associated with strains that harboured changes within the *ramR* gene (Fig. 2A). Ideally, complementation studies with wild-type *ramR* would have been performed to confirm whether the *ramR* changes were directly associated with *ramA* overexpression. However, this was not possible owing to the multidrug-resistant profiles of the different strains.

#### 3.5.2 Promoter (pI and pII) mutations

Unlike previous reports, the majority of the clinical isolates did not harbour any changes within the pI promoter. However, the lack of promoter-associated changes linked to increased *ramA* expression is not unique to this cluster of isolates, as the original *ramA*-overexpressing strain Ecl8MdrI (as reported by George et al. [15]) also does not harbour any changes within the *ramR* gene, the palindrome or the −10 and −35 hexamers at the pI promoter. Only one strain (TS170), which sustained a change within the palindromic sequence (C → T, recognised by RamR) but harboured no changes within the *ramR* gene, was found. Correspondingly, TS170 exhibited a small increase in *ramA* expression (Fig. 2A). Several changes associated with the pII promoter region were located downstream of the second putative palindrome but upstream of the RACE-mapped transcriptional start site of *ramA* (Fig. 1B). Three changes (T206C, G229A and A235T) were consistently identified in four strains (S7, TS152, TS165 and TS261) (Table 3B), all of which overexpressed *ramA.* Of these strains, only S7 and TS261 harboured no changes within the *ramR* gene (Table 3A).

#### 3.5.3 Mutations within the acrR gene and levels of AcrA protein

Of the 27 strains tested, 9 sustained changes within the *acrR* gene. Of these changes, only two (T5N and L214F) have been previously documented [11]. The other nucleotide substitution, but not amino acid change (E147E), was found in a region previously shown to be implicated in AcrA overexpression. One new silent change (P155P, CCC → CCT) was observed. Given that most of the changes observed were silent, it is unlikely that they would have contributed significantly to AcrA levels. Strains TS165, TS173, TS257 and TS293, which significantly overexpressed *ramA*, appear not to produce greater levels of the AcrA protein (Fig. 2B).

## 4 Discussion

Recent studies have linked antibiotic resistance, particularly tigecycline resistance, to increased expression of the *ramA* gene. In this study, we identified in silico that key regulatory features of the *ramA* locus are conserved amongst *Klebsiella*, *Enterobacter*, *Citrobacter* and *Salmonella* spp.

This work also shows that both the RamR and RamA proteins bind the pI and pII promoters specifically, implying the presence of recognition sites for both proteins within these promoter regions. Like other orthologous systems, such as MarRAB and SoxRS [24], binding of purified RamA to the pI and pII promoters indicates that *ramA* autoregulates its own expression. In addition, RamR appears to be able to bind regions that either contain a perfect palindrome or more degenerate palindromic sites (Figs. 1A and 3A). Other TetR-like regulators have been shown to regulate via palindromic binding sites that are identified as lower-affinity binding sites owing to key differences in the palindromic binding sequence.

The present results show that regulation of the *romA*–*ramA* locus is not entirely identical to that observed in *Salmonella* as there are two promoters within the *romA*–*ramA* locus. The presence of these two promoters supports the possibility that the *romA*–*ramA* genes can be regulated independently of each other or as part of an operon (Fig. 2A). Consistent with this genetic arrangement, changes were identified within the pI and putative pII promoter regions in the *ramA*-overexpressing strains (Table 3B). Interestingly, levels of *romA* and *ramA* expression are not linked, supporting the finding that two different promoters control regulation of the *romA*–*ramA* locus (Fig. 2A).

We chose to extend the molecular findings by determining the regulation of *ramA* expression in clinical isolates that were resistant or intermediately susceptible to tigecycline. The results demonstrate that in geographically diverse isolates of *K. pneumoniae*, 18 isolates exhibited tigecycline MICs ≥ 4 μg/mL (Table 3A). A notable example is strain TS257, which exhibits a tigecycline MIC of 16 μg/mL, and where no mutations were observed within the *ramR*–*romAramA* locus but it still overexpressed the *ramA* gene. Two key observations arise from these data: first, *ramA* is not always associated with *ramR*-mediated derepression; and second, decreased tigecycline susceptibility is not always associated with *ramA* expression. We also did not observe any significant mutations within the promoter regions, as has been observed previously [16,17]. An interesting point is that the isolates in this study pre-date the use of tigecycline in the relevant hospitals, which underscores the likelihood that most antibiotics have the propensity to select for changes that result in *ramA* overexpression, a similar situation that is noted for the *marRAB* and *soxRS* systems [24]. The lack of a direct association between *ramA* and *acrA* expression has been noted previously [25] and supports the current data.

Overexpression of transcriptional regulators such as RamA triggers the expression of a multitude of genes that confer pleiotropic phenotypes [26] (Schneiders, unpublished data). Given the varied bacterial response that is mounted with *ramA* overexpression, we should consider the broader implications of these intrinsic resistance mechanisms in the development and persistence of antimicrobial resistance.

## Figures and Tables

**Table 1:** Primer sequences used in the study.

Primer	Sequence
ramAF	5′-AGCCTGGGGCGCTATATT-3′
ramAR	5′-GTGGTTCTCTTTGCGGTAGG-3′
romAF	5′-GAAGCGTAACCAGACGCTGT-3′
romAR	5′-CTGGTCATACTGCCCGTTCT-3′
ramRF	5′-AACTGCAGTCGTCAAGACGATTTTCAATTTT-3′
ramRR	5′-AAAAGTACTAGTGTTTCCGGCGTCATTAG-3′
acrRF	5′-TTAAGCTGACAAGCTCTCCG-3′
acrRR	5′-ACGTAACCTCTGTAAAGTCAT-3′
pIF	5′-GGGCCAGTTTTCTGTT-3′
pIR	5′-ATAGTATCAATCACCTGAGC-3′
pIIF	5′-CTACTTTTTTCCTCACGCAG-3′
pIIR	5′-CCCTGCGGCGCCTTACCA-3′
16SF	5′-GTTACCCGCAGAAGAAGCAC-3′
16SR	5′-CTACGCATTTCACCGCTACA-3′
ramAR1	5′-TTGCAGATGCCATTTCGA-3′
ramAR2	5′-TATCATCAATACGCAGCG-3′
ramAR3	5′-GGGGTACCATAGTATCAATCACCTGAGC-3′

**Table 2:** Minimum inhibitory concentrations (MICs) for 157 clinical isolates.

Antimicrobial agent	MIC (μg/mL)	
	MIC_50_	MIC_90_
Ciprofloxacin	128	>128
Ceftazidime	>128	>128
Tetracycline	>128	>128
Minocycline	32	128
Tigecycline	1	2

MIC_50/90_, MIC for 50% and 90% of the organisms, respectively.

**Table 3A:** Association between RamR and AcrR changes and tigecycline minimum inhibitory concentration (MICs).

Strain	RamR	AcrR	Tigecycline MIC (μg/mL)	Origin
S7	No change	[11]	8	Singapore
S28	15 nt Δ (558–573 bp)	[11]	2	Singapore
S29	7 nt insertion at nt position 561	[11]	16	Singapore
TS152	T119P	No change	4	Turkey
TS165	T119P	GAG440GAA (E147E)	4	Turkey
TS170	No change	No change	2	Turkey
TS173	No change	No change	2	Turkey
TS184	No change	No change	4	Turkey
TS202	H186N, A187E	No change	2	Chile
TS215	E41K	CCC465CCT (P155P)	4	Chile
TS221	No change	GAG440GAA (E147E)	2	Chile
TS238	A19V	GAG440GAA (E147E)	4	Pakistan
TS240	No change	CCC465CCT (P155P)	4	Pakistan
TS245	No change	TTG641TTT (L214F)	4	Pakistan
TS248	No change	ACC14AAC (T5N)	4	Pakistan
TS250	No change	GAG440GAA (E147E)	4	Pakistan
TS251	No change	No change	8	Pakistan
TS257	No change	No change	16	Pakistan
TS259	No change	No change	2	Pakistan
TS261	No change	GAG440GAA (E147E)	4	Pakistan
TS262	I141T	No change	4	Pakistan
TS267	No change	No change	4	Pakistan
TS293	I141T	CCC465CCU (P155P)	4	Pakistan
TS308	W94Stop	No change	4	Pakistan

nt, nucleotide.

**Table 3B:** Changes observed within the pI and pII promoter regions.

Strain	pI	pII
S7	T141C	**T206C**^a^
S28	–	–
S29	–	–
TS 152	–	**T206C**, G229A, A235T
TS 165	–	**T206C**, G229A, A235T
TS 170	T141C, C56T^b^	–
TS 243	T141C	–
TS 245	T141C	–
TS 248	T141C	–
TS 250	T141C	–
TS 251	T141C	–
TS 257	T141C	–
TS 261	T141C	**T206C**, G229A, A235T

a Bold indicates a change within the putative – 35 hexamer sequence upstream of the *romA* gene.

b Indicates a recurring change that occurs in clinical isolates from geographically distinct locations.

## References

[1] Hernandez-Alles S., Alberti S., Alvarez D., Domenech-Sanchez A., Martinez-Martinez L., Gil J. (1999). Porin expression in clinical isolates of *Klebsiella pneumoniae*. Microbiology.

[2] Chevalier J., Pagès J.M., Eyraud A., Mallea M. (2000). Membrane permeability modifications are involved in antibiotic resistance in *Klebsiella pneumoniae*. Biochem Biophys Res Commun.

[3] Ardanuy C., Linares J., Dominguez M.A., Hernandez-Alles S., Benedi V.J., Martinez-Martinez L. (1998). Outer membrane profiles of clonally related *Klebsiella pneumoniae* isolates from clinical samples and activities of cephalosporins and carbapenems. Antimicrob Agents Chemother.

[4] Rice L.B. (2009). The clinical consequences of antimicrobial resistance. Curr Opin Microbiol.

[5] Livermore D.M. (2005). Tigecycline: what is it, and where should it be used?. J Antimicrob Chemother.

[6] Keeney D., Ruzin A., McAleese F., Murphy E., Bradford P.A. (2008). MarA-mediated overexpression of the AcrAB efflux pump results in decreased susceptibility to tigecycline in *Escherichia coli*. J Antimicrob Chemother.

[7] Keeney D., Ruzin A., Bradford P.A. (2007). RamA, a transcriptional regulator, and AcrAB, an RND-type efflux pump, are associated with decreased susceptibility to tigecycline in *Enterobacter cloacae*. Microb Drug Resist.

[8] Ruzin A., Keeney D., Bradford P.A. (2007). AdeABC multidrug efflux pump is associated with decreased susceptibility to tigecycline in *Acinetobacter calcoaceticus*–*Acinetobacter baumannii* complex. J Antimicrob Chemother.

[9] Ruzin A., Keeney D., Bradford P.A. (2005). AcrAB efflux pump plays a role in decreased susceptibility to tigecycline in *Morganella morganii*. Antimicrob Agents Chemother.

[10] Ruzin A., Visalli M.A., Keeney D., Bradford P.A. (2005). Influence of transcriptional activator RamA on expression of multidrug efflux pump AcrAB and tigecycline susceptibility in *Klebsiella pneumoniae*. Antimicrob Agents Chemother.

[11] Schneiders T., Amyes S.G., Levy S.B. (2003). Role of AcrR and RamA in fluoroquinolone resistance in clinical *Klebsiella pneumoniae* isolates from Singapore. Antimicrob Agents Chemother.

[12] Piddock L.J. (2006). Multidrug-resistance efflux pumps—not just for resistance. Nat Rev Microbiol.

[13] Padilla E., Llobet E., Domenech-Sanchez A., Martinez-Martinez L., Bengoechea J.A., Alberti S. (2010). *Klebsiella pneumoniae* AcrAB efflux pump contributes to antimicrobial resistance and virulence. Antimicrob Agents Chemother.

[14] Ma D., Alberti M., Lynch C., Nikaido H., Hearst J.E. (1996). The local repressor AcrR plays a modulating role in the regulation of *acrAB* genes of *Escherichia coli* by global stress signals. Mol Microbiol.

[15] George A.M., Hall R.M., Stokes H.W. (1995). Multidrug resistance in *Klebsiella pneumoniae*: a novel gene, *ramA*, confers a multidrug resistance phenotype in *Escherichia coli*. Microbiology.

[16] Hentschke M., Wolters M., Sobottka I., Rohde H., Aepfelbacher M. (2010). *ramR* mutations in clinical isolates of *Klebsiella pneumoniae* with reduced susceptibility to tigecycline. Antimicrob Agents Chemother.

[17] Abouzeed Y.M., Baucheron S., Cloeckaert A. (2008). *ramR* mutations involved in efflux-mediated multidrug resistance in *Salmonella enterica* serovar Typhimurium. Antimicrob Agents Chemother.

[18] Kallman O., Motakefi A., Wretlind B., Kalin M., Olsson-Liljequist B., Giske C.G. (2008). Cefuroxime non-susceptibility in multidrug-resistant *Klebsiella pneumoniae* overexpressing *ramA* and *acrA* and expressing *ompK35* at reduced levels. J Antimicrob Chemother.

[19] Okusu H., Ma D., Nikaido H. (1996). AcrAB efflux pump plays a major role in the antibiotic resistance phenotype of *Escherichia coli* multiple-antibiotic-resistance (Mar) mutants. J Bacteriol.

[20] Merlin C., McAteer S., Masters M. (2002). Tools for characterization of *Escherichia coli* genes of unknown function. J Bacteriol.

[21] Andrews J.M., BSAC Working Party on Susceptibility Testing (2009). BSAC standardized disc susceptibility testing method (version 8). J Antimicrob Chemother.

[22] Hawser S.P. (2010). Global monitoring of cross-resistance between tigecycline and minocycline, 2004–2009. J Infect.

[23] Bryson K., McGuffin L.J., Marsden R.L., Ward J.J., Sodhi J.S., Jones D.T. (2005). Protein structure prediction servers at University College London. Nucleic Acids Res.

[24] Alekshun M.N., Levy S.B. (1999). The *mar* regulon: multiple resistance to antibiotics and other toxic chemicals. Trends Microbiol.

[25] Ruzin A., Immermann F.W., Bradford P.A. (2008). Real-time PCR and statistical analyses of *acrAB* and *ramA* expression in clinical isolates of *Klebsiella pneumoniae*. Antimicrob Agents Chemother.

[26] Bailey A.M., Ivens A., Kingsley R., Cottell J.L., Wain J., Piddock L.J. (2010). RamA, a member of the AraC/XylS family, influences both virulence and efflux in *Salmonella enterica* serovar Typhimurium. J Bacteriol.

